# Does It work in Clinical Practice? A Comparison of Colonoscopy Cleansing Effectiveness in Clinical Practice Versus Efficacy from Selected Prospective Trials

**DOI:** 10.1093/jcag/gwy070

**Published:** 2019-02-12

**Authors:** Chang (Nancy) Wang, Ruobing Yang, Lawrence Hookey

**Affiliations:** 1 Queen’s University School of Medicine, Kingston, Ontario, Canada; 2 Gastrointestinal Diseases Research Unit, Department of Medicine, Queen’s University, Kingston, Ontario, Canada

**Keywords:** Bowel preparation quality, Split-dose regimen, Efficacy versus effectiveness

## Abstract

**Background:**

Adequate bowel preparation is essential for a high-quality colonoscopy. Many randomized controlled trials (RCTs) have investigated bowel preparation protocols, including split-dose and low-volume regimens. However, RCTs are conducted in an ideal, controlled setting, and translation of trial results to clinical practice is challenging. In this study, we compared the quality of bowel preparations of real-world patients from clinical practice with those enrolled in several prospective trials.

**Methods:**

Bowel preparation quality, defined by the Ottawa Bowel Preparation Scale (OBPS), from four RCTs and one prospective trial were compared with two observational diary studies. Bowel preparations were polyethylene glycol preparation (PEG) or sodium picosulfate plus magnesium citrate (P/MC) taken via traditional or split-dose timing regimen. Age, sex, average number of bowel movements per day, comorbidities, colonoscopy indication and colonoscopy completion rates were also collected.

**Results:**

Patients enrolled in prospective trials had a better OBPS by one point when compared with those in observational clinical practice studies (P<0.049), after controlling for age, sex, colonoscopy indication and type of bowel preparation used. We also found that each 10-year increase in age was associated with a 0.2 point increase in OBPS (P=0.008), and men were associated with a 0.5 increase in OBPS when compared with women P=0.014).

**Conclusion:**

Patients from clinical practice have higher OBPS than prospective trial patients. Increased age and male sex were also associated with increased OBPS. We believe increased patient motivation and education around bowel preparation regimen plays an important role in the success of bowel preparations.

Colonoscopy is essential for the diagnosis of many colonic diseases including colorectal cancer and inflammatory bowel diseases (IBDs). For adequate visualization of the bowel mucosa, patients must take a laxative preparation to wash the bowel of stool and digested food products. However, colonoscopy incompletion rates have been reported anywhere from 4% to 25% (1–8), and poor bowel preparation is an identified risk factor associated with colonoscopy incompletion (9, 10). This is estimated to cost an additional 10% to 20% in health care expenditures for follow-up colonoscopies (11).

There have been many different bowel preparations protocols assessed over the years in randomized controlled trials (RCTs) (12–16). For example, recent guidelines have included a multitude of different preparations including polyethylene glycol (PEG) with electrolyte lavage solution (ELS), low volume PEG, magnesium citrate, sodium phosphate, and mixed preparations (17, 18). In addition to medication used, the timing of administration has also been studied (7, 19, 20). The traditional regimen requires the patient to take both preparation doses in the evening before the colonoscopy, five hours apart. The split-dose regimen requires one dose to be taken the evening before and the second the morning of the colonoscopy. Many studies have shown the split dose regimen to give superior bowel preparations when compared with the traditional dose (14, 19, 21–25), but questions have also been raised regarding patient compliance with respect to the early morning doses involved in the split-dose regimen (26, 27). Additional challenges with bowel cleansing preparation include dietary restriction (usually some version of fluids only for all or a portion of the day before the colonoscopy), ingestion of a large amount of fluids to assist the laxative action (particularly important for osmotic preparations), and timing the ingestion of the medications.

 Randomized controlled trials are the gold standard in medicine to demonstrate intervention efficacy. However, the same characteristics that allow strong internal validity in RCTs may also limit their external validity and translation into a ‘real-world’ clinical setting. Randomized controlled trials are conducted in the ideal study setting of a controlled trial with strict exclusion criteria, with enhanced attention from research associates and often with medications provided through the trial. Factors such as compliance, education and patient motivation can significantly dampen the effect size seen in RCTs (28). Effectiveness or pragmatic studies (28, 29) help determine if the efficacy demonstrated in RCTs translates into comparable effectiveness in the clinical setting. Such studies have been conducted in many fields, ranging from asthma (30) and CPR (31) to ultrasound screening of hepatocellular carcinoma (32). Surprisingly, there have been no effectiveness studies for bowel preparations.

Bowel preparations studies may be particularly vulnerable to poor external validity because of the large role patient compliance plays in outcomes. Poor compliance risk factors include failure to follow instructions, gender and cognitive status (6). The latest surge in studies investigating the effect of various educational interventions (33–35) reinforces the notion that despite positive clinical trials, there is room for improvement in real-life experience. Therefore, studying how effective bowel cleansing regimens are in clinical practice may significantly impact clinical decisions. In this study, we compared the effectiveness of bowel preparation of patients in the clinical setting with those enrolled in several prospective trials in our centre.

## METHODS

### Study Design

This was a retrospective study comparing the effectiveness of colonoscopy bowel preparation regimens in clinical practice with the efficacy determined through prospective trials. Raw data were available from bowel preparation studies published over the last 10 years at the Gastrointestinal Diseases Research Unit of Queen’s University in Kingston, Ontario. Therefore, data were collected from five prospective studies, including four randomized controlled trials (RCTs) (7, 19, 36, 37) and one nonrandomized prospective trial (38), and from two observational prospective studies consisting of routinely booked patients who were asked to provide a diary log on their experience with preparations (21, 39). Further details on these studies are summarized in Table 1. Regimen for each study may be found in Appendix A; all patients included were recruited from outpatient colonoscopy. Age, sex, average number of bowel movements per day, patient comorbidities, indications for colonoscopy, colonoscopy completion rates and the Ottawa bowel preparation score (OBPS) were collected. All endoscopists were trained on use of the Ottawa Bowel preparation score with a post-testing correlation of 0.77 as previously described (7). Data were extracted and collated from existing study records and entered into a single database. Some data reformatting (i.e., classifying average daily bowel movements categorically rather than as continuous variable) was necessary to allow comparison across studies. A total of 1372 patients who underwent outpatient colonoscopy from these seven studies were analyzed. The four different bowel preparation types included a polyethylene glycol plus electrolyte solution preparation (PEG-ELS) or sodium picosulfate plus magnesium citrate preparation (P/MC) taken in a traditional or split-dose regimen. See Appendix A for patient instructions and specific regimen for each study.

**Table 1.** Baseline characteristics of the 7 studies analyzed

	Prospective trials				Clinical practice diary studies		
	(Fleming et al., 2012)	(Melicharkova et al., 2013)	(Hookey & Vanner, 2009)	(Fowler et al., 2009)	(Flemming et al., 2015)	(Arya et al., 2014)	(Vanner & Hookey, 2011)
Patient characteristics	N = 236 (%)	N = 108^a^ (%)	N = 213 (%)	N = 49 (%)	N = 109^a^ (%)	N = 557 (%)	N = 100
Age, y, mean (SD)	56.3 (11.1)	57.0 (12.4)	54.6 (9.8)	75 (3.5)	62.7 (13.8)	59.2 (11.2)	60.2 (11.9)
Age ≥ 70 y	20 (8.5)	12 (11.1)	14 (6.6)	49 (100.0)	41 (37.6)	102 (18.3)	22
Female Sex	127 (53.8)	60 (55.6)	106 (49.8)	24 (49.0)	60 (55.0)	291 (52.2)	58
Avg bowel movement (SD)	1.5 (1.0)	1.6 (1.3)	1.1 (0.5)	1.5 (0.8)	1.8 (1.5)	1.7 (1.8)	1.7 (1.1)
Comorbidities^b^	16 (6.8)	Not collected	5 (2.3)	9 (18.4)	Not collected	243 (43.6)	16
Bowel preparation							
PEG, traditional	0	0	0	0	49 (45.0)	14 (2.5)	0
PEG, split-dose	0	0	0	0	60 (55.0)	158 (28.4)	0
P/MC, traditional	117 (49.6)	68 (63.0)	104 (48.8)	25 (51.0)	0	218 (39.1)	34
P/MC, split-dose	119 (50.4)	40 (37.0)	0	23 (46.9)	0	150 (26.9)	66
Other	0	0	109 (51.2)	0	0	9 (1.6)	0
Indication for colonoscopy							
Screening^c^	132 (55.9)	80 (74.1)	138 (64.8)	9 (18.4)	15 (13.8)	238 (42.7)	30
FOBT+	16 (6.8)	28 (25.9)	7 (3.3)	7 (14.3)	5 (4.6)	47 (8.4)	13
IBS, Crohns, colitis	5 (2.1)	0	9 (4.2)	0	10 (9.2)	40 (7.2)	54
Bowel habit change, NYD	14 (5.9)	0	9 (4.2)	2 (4.0)	10 (9.2)	23 (4.1)	9
Anemia	1 (0.4)	0	2 (0.9)	1 (2.0)	26 (23.9)	22 (3.9)	6
Diarrhea	8 (3.4)	0	7 (3.3)	1 (2.0)	13 (11.9)	1 (0.2)	6
BRBPR	16 (6.8)	0	11 (5.2)	3 (6.1)	12 (11.0)	11 (2.0)	6
Known polyp	41 (17.4)	0	29 (13.6)	25 (51.0)	17 (15.6)	55 (9.9)	22
Nausea/vomiting	1 (0.4)	0	0	0	1 (0.9)	0	0
Abdominal pain	0	0	1 (0.5)	1 (2.0)	0	3 (0.5)	2
Unknown	2 (0.8)	0	0	0	0	117 (21)	0

All values are number (percentage) of patient characteristic in each study, unless otherwise specified.

Abbreviations: FOBT, fecal occult blood test; IBS, irritable bowel syndrome; NYD, not yet diagnosed; BRBPR, bright red bleeding per rectum

^a^Only included patients randomized to non-breakfast study arm.

^b^Pico Timing Study, Original Pico Study and Pico in Elderly study only looked at diabetes mellitus, where the Diary study looked at DM, chronic obstructive pulmonary disease (COPD) and kidney disease.

^c^Screening includes colonoscopy indicated through positive family history.

#### Normal bowel habit

All studies collected the normal bowel habits as average number of bowel movements (BM) per day with the exception of Vanner and Hookey (2011), where the data were represented using categories <0.33 BM/day, 0.33 to 1 BM/day, 1 to 3 BM/day, >3 BM/day, or irritable bowel syndrome (IBS)/alternating.

#### Comorbidities

Studies were highly variable in reporting patient comorbidities. The observational studies by Vanner and Hookey (2011) and Arya et al. (2014) indicated patients were comorbid if they were managing diabetes mellitus, chronic obstructive pulmonary disease or chronic kidney disease. Hookey and Vanner (2009), Flemming et al. (2012) and Fowler et al. (2009) only included data on the comorbidity of diabetes mellitus. No data were available on patient comorbidities in the studies conducted by Melicharkova et al. (2013) and Flemming et al. (2015).

#### Indication for colonoscopy

Asymptomatic indications included screening, positive family history and fecal occult blood test positive. Symptomatic indications for colonoscopy included irritable bowel syndrome (IBS), inflammatory bowel disease (IBD), bowel habit change not yet determined, anemia, diarrhea, bright red blood per rectum, known polyp, nausea/vomiting, abdominal pain and *Clostridium difficile* infection (one patient in study [39] classified under diarrhea). See Table 1 for indications of colonoscopy across the seven studies analyzed in this article.

### Outcomes

The primary outcome was the difference in efficacy of colon cleansing assessed using the Ottawa bowel preparation score (40) between the prospective trial data and the clinical practice data. The secondary outcome was colonoscopy completion rate. Covariates studied included age, sex, average number of bowel movements per day, comorbidities and indications for colonoscopy.

One prospective study focused on patients over the age of 70. Anticipating that this study will skew the prospective trials patient demographic, data analysis was performed both including and excluding this study.

### Statistical Analysis

We used a two-sample *t* test to compare the Ottawa bowel preparation scores between treatment arms within trials. The prospective trials and clinical practice studies were pooled using a linear mixed effects model with a random intercept and random treatment effect to account for potential heterogeneity in the Ottawa bowel preparation scores and the treatment effects between studies. Finally, the Ottawa preparation scores obtained from the prospective trials were compared with those obtained from the clinical observational studies using a linear mixed effects model with random intercepts and treatment effects and controlling for age, sex, indication and bowel preparation method. We used restricted maximum likelihood with denominator degrees of freedom estimated by the Satterthwaite method, as implemented in the mixed procedure of SAS Version 9.4.

## RESULTS

Data were collected and analyzed from 715 patients from the five prospective trials, and 657 patients from the two observational prospective studies. Patient ages ranged from 19 to 98 years and were instructed to use a variety of bowel preparation types. Indications for colonoscopy ranged from asymptomatic indications such as screening or a positive fecal occult blood test (FOBT) result to symptomatic indications such as irritable bowel syndrome, Crohn’s disease, ulcerative colitis, anemia, diarrhea, bright red bleeding per rectum (BRBPR), known polyps, nausea/vomiting, abdominal pain, *Clostridium difficile* infection or change in bowel habit. Table 1 summarizes the baseline characteristics of patients from each of the seven studies.

We then pooled prospective trial data and the clinical practice data (Table 2). After excluding the trial specifically looking at bowel preparations in the elderly, the mean age (SD) in the pooled prospective trials was 56.9 (11.7%), and the number of patients with age greater than 70 was 87 (12.2%). In comparison, the mean age was 59.3 (11.3%) and 18.9% of patients were aged 70 and older (P<0.0001) in the pooled clinical practice data. Six per cent of patients had comorbidities in the prospective trials compared with 39.4% in the pooled clinical practice data.

**Table 2.** Summary of baseline patient characteristics and the OBPS between pooled prospective trials and clinical practice diary studies data

Patient characteristic	Pooled prospective trials	Pooled clinical practice diary trials
	N = 715 (%)	N = 657 (%)
Age, y, mean (SD)	58.2 (12.2)^a^	59.3 (11.3)
Age > 70 y	136 (19.0)^a^	124 (18.9)
Female sex	377 (52.7)	349 (53.1)
Avg bowel mvmt per day, mean (SD)	1.4 (1.0)	1.7 (1.7)
Comorbidities	30 (6.0)^b^	259 (39.4)
Indication for scope		
Asymptomatic	439 (61.4)	328 (49.9)
Symptomatic	274 (38.3)	211 (32.1)
Scope incomplete	42 (6.3)	11 (1.7)
OBPS, mean (SD)	4.8 (2.7)	5.7 (2.9)

All values are number (percentage) of patient characteristic in each study, unless otherwise specified.

^a^After excluding the prospective trial specifically looking at bowel preparations in the elderly, the mean age (SD) was 56.9 (11.7) and the number of patients with age >70 years was 87 (12.2%), both significantly different form the clinical practice diary studies with P < 0.0001.

^b^Since comorbidities data was only available from Hookey and Vanner, 2009, Fowler et al., 2009, and Flemming et al., 2012. This amounted to N = 498 for the purposes of calculating comorbidities.

Since PEG traditional and split-dosing bowel preparations were only studied in one prospective study and one observational study, we concentrated instead on the effect of P/MC bowel preparations. Table 3 provides the four prospective studies and two diary studies with estimates pooling the results across studies. Both the split-dose and traditional bowel preparations methods had lower mean OBPS values in the prospective trials when compared with the diary studies. The difference between the traditional and split-dosing varied across the three prospective studies comparing the bowel preparations, with only the largest study demonstrating a significant benefit of the split-dose method over the traditional dosing (P<0.0001). Neither of the observational studies suggested a significant difference between the two bowel preparation methods.

**Table 3.** Comparing Ottawa bowel preparation scores between traditional and split-dose P/MC bowel preparations in individual and pooled prospective trials and clinical practice diary studies

Type	**Study**	Traditional dose		Split-dose		Difference		
		**n**	**Mean (95% CI)**	**n**	**Mean (95% CI)**	**Mean (95% CI)**	df*	P value
Prospective trials	(Flemming et al., 2012)	109	5.5 (5.0 to 6.0)	113	4.1 (3.6 to 4.5)	1.5 (0.8 to 2.1)	220	<0.0001
	(Fowler et al., 2009)	24	4.9 (3.9 to 6.0)	23	5.0 (3.9 to 6.1)	-0.1 (-1.6 to 1.3)	45	0.863
	(Melicharkova et al., 2013)	56	4.7 (4.0 to 5.5)	36	4.4 (3.4 to 5.3)	0.3 (-0.8 to 1.5)	90	0.569
	(Hookey & Vanner, 2009)	100	5.0 (4.5 to 5.5)					
	Pooled Mixed model		5.1 (4.5 to 5.7)		4.3 (3.6 to 5.0)	0.8 (-0.1 to 1.6)	4.4	0.080
Clinical practice diary studies	(Arya et al., 2014)	216	6.1 (5.8 to 6.5)	149	6.2 (5.7 to 6.6)	0.0 (-0.6 to 0.5)	363	0.927
	(Vanner & Hookey, 2011)	34	5.2 (4.1 to 6.3)	61	5.0 (4.3 to 5.7)	0.3 (-0.9 to 1.5)	93	0.663
	Pooled Mixed model		5.7 (0.7 to 11.2)		5.6 (-0.1 to 11.3)	0.0 (-0.5 to 0.7)	458	0.860
(Prospective trials) – (Clinical practice studies) adjusted for age, sex, and indication			0.7 (0.4 to -1.7)		1.4 (0.3 to 2.5)	0.7 (-1.2 to 2.6)		0.266

*df, degrees of freedom.

Finally, using a multivariable mixed effects model, we found that greater age, male sex, and results of observational clinical studies (versus a prospective trial) were all associated with worse (higher) Ottawa bowel preparation scores (see Table 4). Each decade increase in age was associated with a 0.2 increase in OBPS (P=0.008), male patients were associated with a 0.5 point increase in OBPS (P=0.014), and patients in the observational studies were associated with a 1.0 point higher OBPS on average when compared with the prospective trials (P=0.049).

**Table 4.** Mixed effect multiple regression showing the effect of age, sex, colonoscopy indication, and study type on OBPS

Variable	Estimate (95% CI)	P value
Age (per decade)	0.2 (0.0 to 0.4)	0.008
Male vs. female	0.5 (0.1 to 0.8)	0.014
Colonoscopy Indication **(asymptomatic vs. symptomatic)**	0.2 (-0.2 to 0.6)	0.322
Prospective study vs. Clinical Practice Diary Study	-1.0 (-2.0 to 0.0)	0.049

Further analyses of the split-dose versus traditional P/MC populations in clinical practice showed no significant difference in baseline characteristics. The average ages for split-dose and traditional P/MC populations were 59.2 and 59.3 years (P=0.91), respectively; the average bowel movements per day were 1.37 and 1.46 (*P*=0.23), respectively. Furthermore, split-dose P/MC regimens were not selectively chosen for morning versus afternoon cases, suggesting time from completion of bowel preparation and procedure start (e.g., runway time) did not differ between split-dose and traditional P/MC regimens in clinical practice.

## Discussion

This is the first study evaluating the differences in prospective trials and clinical practice regarding bowel preparation for colonoscopy. Patients in clinical practice tended to have worse colon cleansing (higher OBPS) than prospective trial patients after controlling for patient age, sex, indication of colonoscopy and bowel preparation method. In our study, prospective trial patients tended to be younger than real-world patients, possibly had fewer comorbidities and had higher average daily bowel movements, probably related to significant constipation being an exclusion criterion for many trials. An additional interesting finding was that scope incompletion rates were higher in prospective trials, 6.3% versus 1.7%. This may be explained by the greater proportion of asymptomatic screening indications in the pooled prospective trials when compared with clinical practice, 61.4% versus 49.9% (Table 2). While poor preparations are often aborted in favour of rescheduling when the indication is asymptomatic screening, endoscopists may be more inclined to complete an examination despite a suboptimal preparation to arrive at a diagnosis in clinical practice. Finally, we found that older patients, male sex and patients of the observational studies when compared with prospective trials were each associated with worse OBPS after controlling for other possible confounding variables. Though our results also suggest that the benefit of the split-dose P/MC when compared with traditional dosing reported in prospective trials did not translate to the observational studies (difference in OBPS of 0.8 versus 0.0), we did not have enough power to demonstrate a significant difference.

We postulate that the superior bowel cleansing seen in prospective trials when compared with clinical practice can be explained by the difference in study design. Prospective trials, exemplified by RCTs, are conducted in a controlled setting designed to study one major hypothesis. This requires strict inclusion and exclusion criteria, involves a clinical research associate giving personalized teaching to each subject and being available for questions after the fact, and, in our study and others, often results in a study population that is younger and healthier than the general population (29). The current study shows that this seems to translate to a better bowel preparation.

Another striking signal was that split-dose P/MC regimen in the clinical practice did not impact bowel preparation quality (5.7 versus 5.6, *P*=0.86), though our study was not specifically powered to detect a difference between these split-dose and traditional dose bowel preparations. While split-dose bowel regimen has been shown to be superior across both high- and low-volume bowel preparations in meta-analyses of randomized controlled trials (15, 24, 41), few studies have taken an observational approach to examining how these preparations translate to everyday clinical practice. The few non-RCT observational studies still use strict inclusion and exclusion criteria, which are a poor representation of daily practice (42, 43). We postulate that the split-dose regimen does likely result in superior bowel preparations when compared with the traditional dosing but that decreased motivation, education and compliance have overshadowed the results in clinical practice. Patient noncompliance and poor fluid intake with bowel preparations are strong predictors of poor bowel preparations (44), and it has been speculated that the split-dose regimen may be associated with decreased compliance due to the requirement of early morning doses (27). In the controlled prospective trial setting, significant effort is spent toward giving patients clear instructions for the bowel preparation and fluid intake to optimize compliance. Patients are also encouraged to contact designated research assistants if they have any concerns or questions during the process. Finally, patients may show increased compliance when they are aware that they are part of a research study. In comparison, the clinical practice observational studies simply asked participants to complete a diary of average bowel movements of the week before colonoscopy, fluid intake and timing of bowel preparation intake.

We believe that increased patient education is the key to translating the proven efficacy of bowel preparation regimen of clinical trials into the real world by facilitating patient compliance and adherence. Similar conclusions regarding the importance of dietary education in bowel preparation were raised in the study by Sharara et al. (2016), where real life versus prospective trials were compared. This study showed that while obesity presents as a risk factor for poor bowel preparation in trials of retrospective clinical practice, it is not reflected in prospective controlled trials (45). They suggested that obesity alone was not a direct risk factor of poor bowel preparation but that the difference can be attributed to higher compliance and adherence rates to pre-endoscopy dietary instructions in clinical trials. Our comparison study also helps to stress the importance of patient education in improving patient compliance and bowel preparation quality. Education targets include but are not limited to bowel regimen instructions, fluid intake and risks for poor visualization/repeat colonoscopy with poor bowel preparations.

The nature of the data collection and collation in a mixed group of studies such as these leads to limitations in the study. Our results showed that a greater proportion of clinical practice patients have comorbidities than the pooled prospective trials data, but the significance of this result is limited by the difference in the way comorbidities were defined across the studies and incomplete data. Three of the five prospective trials only recorded diabetes mellitus (7, 19, 38); the two others did not include information on patient comorbidities at all. The two observational studies (21, 39) recorded the presence of diabetes mellitus, chronic obstructive pulmonary disease or chronic kidney disease as positive for comorbidities. While our results are inconclusive due to the nature of the data collection, decreased comorbidities in more uniform RCT population when compared with the general population is a well-documented phenomenon (28, 29). In addition, we have inadequate participants to draw conclusions regarding PEG dosing regimen and incomplete data on comorbidity incidence. We are the first group to find the very interesting result that split-dose P/MC regimen was not associated with better bowel preparations when compared with the traditional dose regimen in the observational studies; however, this conclusion is at risk for a type 1 error because it is the result of subgroup analysis. Finally, all data were collected from a single centre, leading to limitations in the generalizability of our results. As data sharing becomes more readily accessible, comparison of trials across multiple centres is a promising area for future studies.

In conclusion, we have shown that patients in clinical practice have poorer bowel preparations than patients enrolled in a prospective trial or RCT. The split-dose P/MC regimen was not associated with a better OBPS when compared with the traditional dose in the observational studies. This brings to light the need for further observational studies on the effectiveness of split-dose regimen in clinical practice. We believe that this disparity can largely be explained by the increased motivation and compliance in RCT patients, stressing the importance of patient education when prescribing bowel preparations for colonoscopies.

## **Appendix A**. Bowel regimen and patient instructions for the 7 studies analyzed.

**Table:**

**Paper**	**Bowel Regimen**	**Patient Instructions**
**Prospective Trials**				
Hookey and Vanner, 2009	1-PSLx* 1700 and 2200 evening prior + 10mg Bisacodyl po days 2, 3 pre-colonoscopy 2-PSLx 1700 and 2200 alone evening prior 3-45mL oral NaPhos 1700 and 2200 evening prior	CF only day prior 3-4L of Gatorade or similar evening prior
Fowler et al., 2009	All: PSLx mixed in 150–200 mL H2O + 10mg Bisacodyl po days 2, 3 pre-colonoscopy ^+^Before 11am: (traditional)1 package PSLx 1700 then 2200 After 11am: (split-dose) 1 package PSLX day prior 1900, then 5h prior to procedure	4L CF including Gatorade up to 2h before procedure
Fleming et al., 2012	ALL +10mg Bisacodyl days 2, 3 pre-colonoscopy 1-PSLx at 5pm then 11pm day before 2-PSLx at 7pm evening prior, then 4h prior to colonoscopy	CF only day prior 3–4 L of Gatorade or similar
Melicharkova et al., 2013	1-LRB no later than 10am with CF after 2-CF until 2h prior to colonoscopy 10mg Bisacodyl @1700 day 2, 3 pre-colonoscopy then 2x PSLx Before 11am: 2x at 2200 day prior After 11am: 2x at 0600 day of colonoscopy	
Fleming et al., 2015	1-LRB + CF day prior to colonoscopy 2-CF diet alone ALL: 4L PEG-ELS Before 11am: (traditional), 4L over 1-3h starting 1900 After 11am: (split-dose) 2L over 1h at 1900, 2L over 1h 4 hours prior to colonoscopy	LRB – low reside breakfast no later than 10am with CF only after CF up to 2h prior to colonoscopy
**Clinical Practice Diary Studies**				
Vanner and Hookey, 2011	PSLx + 10mg Bisacodyl at 1800 days 2,3 pre-colonoscopy Before 11am: (traditional) 1 package PSLx 1700 then 2200 After 11am: (split-dose) 1 package PSLx evening prior 1900, then 0600 day of	CF day prior, 4L Gatorade or similar day prior until leaving home Diary record average BM x 1wk, times of preparation, times of new BM, amount of sports drink + overall fluids
Arya et al., 2014	Bowel prep chosen by individual gastroenterologists, PEG or PSLx + bisacodyl (10mg days 2, 3 prior to colonoscopy) Before 11am: 1 package PSLx 1700 then 2200, OR 4L PEG over 1-3h starting 1900 After 11am: 1 package PSLx evening prior 1900 and 0600 day of. OR 2L PEG over 1h at 1900 on day prior and 2L L over 1h 4 hours prior to colonoscopy	

Abbreviations: PSLx, pico-salax (picosulfate, magnesium oxide, citric acid); LRB, low residue breakfast; CRD, clear residue diet; CF, clear fluids; po, by mouth ^+^As per protocol in the current centre, split-dosing was used for all patients with colonoscopy appointments scheduled after 11:00 am and traditional dosing was used for appointments before 11:00 am. Individuals living far distances could not tolerate split-dose schedule.
